# Supplementary material for: Effects of timing of cord clamping on neonatal hemoglobin and bilirubin levels in preterm and term infants—A prospective observational cohort study
Source: PLoS One. 2024 Jan 2;19(1):e0295929. doi: 10.1371/journal.pone.0295929 (PMC10760750; doi:10.1371/journal.pone.0295929)
Supplement: S1 Data — (DOC) [file pone.0295929.s001.doc]

ANNEXURE 1:

CASE RECORD

**S. No.**

**Obstetric history**

• Name:

• Age:

• UHID No:

• Age at conception:

• LMP:

• Brief history of previous pregnancies:

**Investigations**

• Blood Group:

• Hb:

• TLC:

• Platelet Count:

• OGTT:

• TSH:

• HIV:

• HBsAg:

• Anti HCV:

• VDRL:

• Urine R/M:

• Antenatal ultrasonography report:

**Mode of delivery:**

**Gestational age:**

**During delivery:**

Time between delivery of newborn and cord clamping by the obstetrician:

**Neonatal outcomes**

Cry immediately after birth: Yes No

APGAR scores at 1min, 5 mins, 10 mins:

Heart rate at 10 mins:

Respiratory rate at 10 mins: Retractions/grunting/nasal flaring:

Oxygen saturation at10 mins:

Birth weight:

AGA/SGA/LGA:

Admission in a NICU:

At 48 hrs

Hb: Hematocrit:

RBC count:

MCV:

RDW:

Platelet count:

Total serum bilirubin: Direct serum bilirubin: Indirect serum bilirubin:
