# Supplementary material for: Effects of timing of cord clamping on neonatal hemoglobin and bilirubin levels in preterm and term infants—A prospective observational cohort study
Source: PLoS One. 2024 Jan 2;19(1):e0295929. doi: 10.1371/journal.pone.0295929 (PMC10760750; doi:10.1371/journal.pone.0295929)
Supplement: Attachment — Submitted filename: Response to Reviewers (1).doc [file pone.0295929.s002.doc]

We thank reviewer for their constructive feedback to improve the manuscript. The detailed answers to reviewer’s queries are given below:

**Reviewer 1**: I congratulate team for conducting such good study. Sample size is adequate and this study provide new insight about the optimal time of cord clamping as DCC is being recommended for all term new-born

Answer: We thank reviewer for his encouraging comments

**Reviewer 2**

1. “The present observational study was conducted to determine the optimal time of DCC clamping in preterm and term infants that maximize benefits without increasing the risk of polycythemia or neonatal hyperbilirubinemia.” This aim is fairly broad. Was there any specific pre-specified hypothesis?

**Answer** – We hypothesized that increasing the duration of cord clamping might improve early haematological parameters without any untoward effects (Page 3; lines 3,4) .

2. Before embarking on the study the obstetric team was consulted and the importance of DCC was elaborated. They were requested to practice DCC of at least ≥30 seconds at their discretion in an individual case.” I am curious to know if this is the total extent of the advice given. Were the obstetric team aware that the purpose of the study was to compare groups based on time to cord clamping after birth? Or given any other advice on how much beyond 30 seconds they should target? There is fairly substantial risk of bias here, as there may have been other clinical factors that influenced the obstetrician’s chosen DCC time, which could also impact outcomes. If the obstetricians were aware of the study design, they could (consciously or sub-consciously) have adjusted their approach to time of cord clamping accordingly

**Answer-** The obstetrician team emphasized the importance and benefits of delayed cord clamping at 30 seconds or more. However, the obstetrician team is not aware of the purpose of the study and the 4 groups mentioned in this study. Thus, the question of change in approach would not arise(Page 6; lines1-3).

3. According to our institutional protocol, after delivery, Oxytocin was given” To clarify: was oxytocin given after the infant was born but prior to cord clamping, or after both birth and cord clamping were complete.

**Answer-** According to our institutional protocol, oxytocin was given at birth after infants was born but prior to cord clamping so that other component of 3^rd^ stage are labor was not affected(Page 6; lines 14,15).

4. Was the study prospectively registered in a clinical trials registry? Has a STROBE checklist been prepared?

**Answer** - The study was not registered in the Clinical Trials Registry because it was not a randomized controlled trial. It is not mandatory for a cohort study to register it. However, the STROBE checklist was prepared and submitted to the PLOS.

5. Can the authors provide a copy of the data collection sheet used for the study? Were any other data points collected beyond those included in the baseline data table and the laboratory test results? I ask this because there are specific outcomes that I would consider important to collect based on the study aim, which is to identify the DCC time that can “maximise benefits without increasing risk of polcythemia or hyperbilirubinaemia”. These last two are addressed, but the benefits are not. The authors mention several relevant outcomes in the introduction: temperature/hypothermia, IVH, NEC, sepsis, mortality, need for transfusion, neurodevelopment. The last outcome is unlikely to feasible, but the others would be. The study aim cannot be achieved if none of these were assessed.

**Answer-** We are sharing data collection form. Here, We studied optimal timing of delayed cord clamping to maximize benefits without any untoward effect in terms of haematological parameters . The many outcomes mentioned above related to very preterm infants but in our study we had mixed population where preterm and term infants both studied and our one of exclusion criteria was infants with gestational age < 28 weeks Gestational age( at end of letter and also attached as additional information) .

6. It does not appear that any primary outcome was identified a priori – is this correct?

**Answer**- The primary outcome was the levels of haemoglobin, hematocrit, and bilirubin at 48 hours of life. Primary outcome was mentioned in manuscripts (Page 5; line 10,11) .

7. Was there any specific reason for the time frame chosen for the study? It could still have been possible to estimate how well the study was powered if a primary outcome had been chosen and an estimate of patient numbers was available.

**Answer:**- The time frame was chosen as arbitrarily. Formal sample size was not calculated because this was a time bound study for 17 months.

8. There are a number of preclinical studies indicating that the early physiological benefit of DCC is not dependent upon a fixed time period, but on whether lung aeration has been achieved prior to cord clamping (see Bhatt 2013, Polglase 2015, and others). Clinical studies have been conducted utilizing this approach, ‘physiological-based cord clamping’ (work by Knol, Brouwer, Badurdeen, the recently completed ABC3 trial and VentFirst trials). I would expect this concept to have been discussed by the authors. It would be interesting to know more about the time of regular spontaneous breathing in this population, or even just the respiratory component of the APGAR scores. This is one reason I asked about other data points that were collected above.

**Answer:-** We did study only on spontaneously breathing infants. But we did not record actual time of spontaneous breathing. The child who was not able to breathe at birth or required any type of resuscitation at birth as per the NRP 2020 guidelines was excluded from the study (Page6; lines 3,4,5) .

**ANNEXURE 1:**

**CASE RECORD (data collection form)**

**S. No.**

**Obstetric history**

• Name:

• Age:

• UHID No:

• Age at conception:

• LMP:

• Brief history of previous pregnancies:

**Investigations**

• Blood Group:

• Hb:

• TLC:

• Platelet Count:

• OGTT:

• TSH:

• HIV:

• HBsAg:

• Anti HCV:

• VDRL:

• Urine R/M:

• Antenatal ultrasonography report:

**Mode of delivery:**

**Gestational age:**

**During delivery:**

Time between delivery of newborn and cord clamping by the obstetrician:

**Neonatal outcomes**

Cry immediately after birth: Yes	No

APGAR scores at 1min, 5 mins, 10 mins:

Heart rate at 10 mins:

Respiratory rate at 10 mins: Retractions/grunting/nasal flaring:

Oxygen saturation at10 mins:

Birth weight:

AGA/SGA/LGA:

Admission in a NICU:

At 48 hrs

Hb: Hematocrit:

RBC count:

MCV:

RDW:

Platelet count:

Total serum bilirubin: Direct serum bilirubin: Indirect serum bilirubin:
